# Hepatic Portal Venous Gas in a Patient With an Intragastric Balloon: A Case Report

**DOI:** 10.7759/cureus.117813

**Published:** 2026-10-12

**Authors:** Sara Eltayeb, Banneh AlAnsari, Khaled Bamakhrama

**Affiliations:** 1 Gastroenterology, Mohammed Bin Rashid University of Medicine and Health Sciences, Rashid Hospital, Dubai, ARE; 2 Gastroenterology, Hepatology, and Endoscopy, Rashid Hospital, Dubai, ARE

**Keywords:** acute gastroenteritis, bariatric medicine, gastric mucosal injury, hepatic portal venous gas, intragastric balloon

## Abstract

Intragastric balloon (IGB) placement is increasingly used as a minimally invasive intervention for weight loss in patients with obesity. While generally safe, IGBs can lead to various gastrointestinal complications. We present a unique case of incidental hepatic portal venous gas (HPVG) in a young woman with an IGB, following an episode of acute gastroenteritis. The patient was managed conservatively with supportive care and removal of the balloon, leading to complete recovery. This case highlights the need to recognize HPVG as a rare but possible finding in patients with IGBs, emphasizing the importance of appropriate diagnosis and individualized management based on the underlying etiology.

## Introduction

Obesity remains a major global health challenge, with rising prevalence and substantial associated morbidity. Lifestyle modification alone often produces limited and poorly sustained weight loss. Bariatric surgery is effective, but many eligible patients are unwilling to undergo it or are poor surgical candidates. Endoscopic bariatric therapies have therefore emerged as minimally invasive options to fill this gap [1].

The intragastric balloon (IGB) is one of the most widely used endoscopic bariatric therapies. It is a space-occupying device that promotes early satiety and reduces food intake, partly by delaying gastric emptying [1]. Most systems are placed and removed endoscopically and filled with saline, although swallowable and adjustable devices also exist [1]. Balloons usually stay in place for about six months, although some adjustable devices are licensed for longer durations [1]. IGBs are generally indicated for adults with a body mass index (BMI) of 30-40 kg/m² who have not achieved adequate weight loss with lifestyle measures, combined with a supervised diet and behavioral program [1]. Pooled data show a mean total body weight loss of approximately 13% at six months [2]. Contraindications include previous gastric surgery, a large hiatal hernia, active peptic ulcer disease, coagulopathy, and pregnancy [1].

IGB placement is technically simple and generally well tolerated. Adverse events range from common, self-limiting symptoms such as nausea, vomiting, and abdominal pain to rare but serious complications. These include balloon migration with bowel obstruction, acute pancreatitis, and gastric or esophageal perforation [1-3]. Several of these, particularly persistent vomiting, gastric distension, and mucosal injury, are also recognized risk factors for hepatic portal venous gas (HPVG) [4,5].

HPVG is an uncommon radiologic finding defined as gas within the portal venous system and its intrahepatic branches. It was classically regarded as an ominous sign of mesenteric ischemia and bowel necrosis, which carry high mortality [4,5]. With the widespread use of computed tomography (CT), however, it is increasingly detected in benign, non-ischemic conditions [5,6].

To our knowledge, HPVG in a patient with an IGB is rarely reported. In that case, migration of a Spatz balloon catheter into the duodenum led to severe acute necrotizing pancreatitis, gastric ischemia, and HPVG, which required surgical intervention [7]. Here, we describe HPVG in a patient with an IGB in place who also had acute gastroenteritis, recurrent vomiting, gastric distension, and severe erosive gastropathy. Unlike the previous case, the course was benign, and the HPVG resolved with conservative management. We discuss the likely multifactorial mechanism and how much the balloon may have contributed.

## Case presentation

A 27-year-old woman with no significant past medical history except for an IGB placed 11 months prior presented with a three-day history of epigastric pain, nausea, repeated vomiting of gastric contents, and loose stools. Symptoms began after consuming food from an external source. She was afebrile and hemodynamically stable on presentation. Abdominal examination revealed a soft abdomen with mild tenderness in the epigastric and umbilical regions. Initial laboratory investigations showed leukocytosis with neutrophilia, while C-reactive protein, amylase, lipase, and renal function tests were within normal limits. Stool culture was unremarkable, and urinalysis revealed pyuria with positive leukocyte esterase (Table 1).

**Table 1:** Laboratory investigations

Laboratory parameter	Patient value	Reference range
White blood cell count	17.7 × 10³/µL	3.6–11.0 × 10³/µL
Hemoglobin	9.4 g/dL	12.0–15.0 g/dL
Platelet count	350 × 10³/µL	150–410 × 10³/µL
C-reactive protein	0.7 mg/L	<0.5 mg/L
Total bilirubin	<0.15 mg/dL	0–1.2 mg/dL
Alkaline phosphatase	66 U/L	35–104 U/L
Alanine aminotransferase (ALT)	7 U/L	0–31 U/L
Aspartate aminotransferase (AST)	17 U/L	0–32 U/L
Albumin	4.1 g/dL	3.9–5.0 g/dL
Lipase	32 U/L	13–60 U/L
Amylase	53 U/L	28–100 U/L
Lactate	1.09 mmol/L	0.5–2.2 mmol/L
D-dimer	0.74 µg/mL FEU	<0.50 µg/mL FEU
Urine routine	Leukocyte estarase positive	Negative
Stool culture	No growth	No growth

Contrast-enhanced CT of the abdomen demonstrated a distended stomach with the balloon in situ but intact. Notably, diffuse gas was observed within the portal vein and along the right gastroepiploic vein (Figures 1, 2).

**Figure 1:**
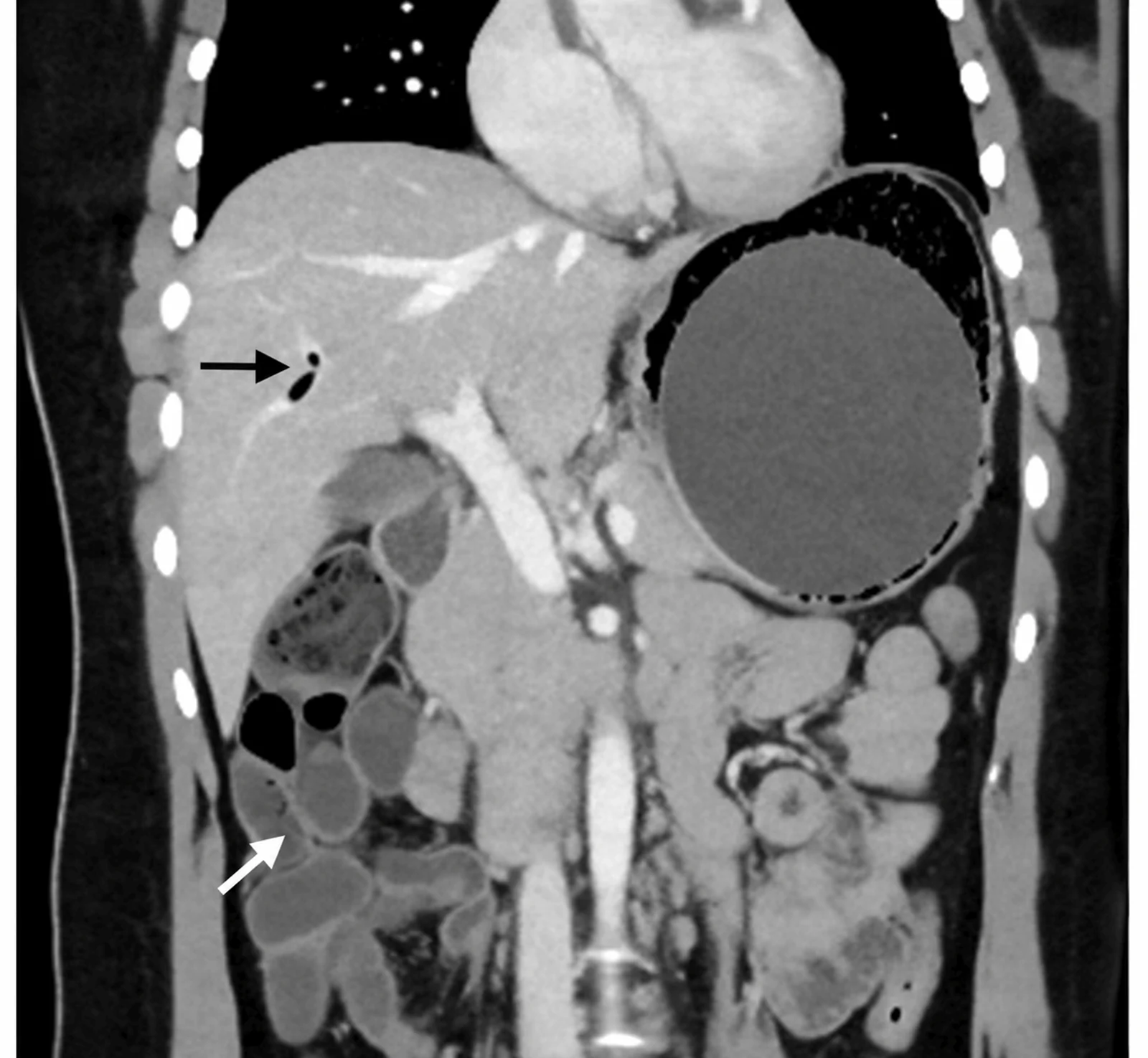
Coronal contrast-enhanced CT of the abdomen demonstrating hepatic portal venous gas (black arrow), with no features of bowel ischemia, as evidenced by preserved bowel-wall enhancement (white arrow) and absence of pneumatosis intestinalis The black arrow indicates air within the portal venous system, and the white arrow shows normal bowel wall enhancement.

**Figure 2:**
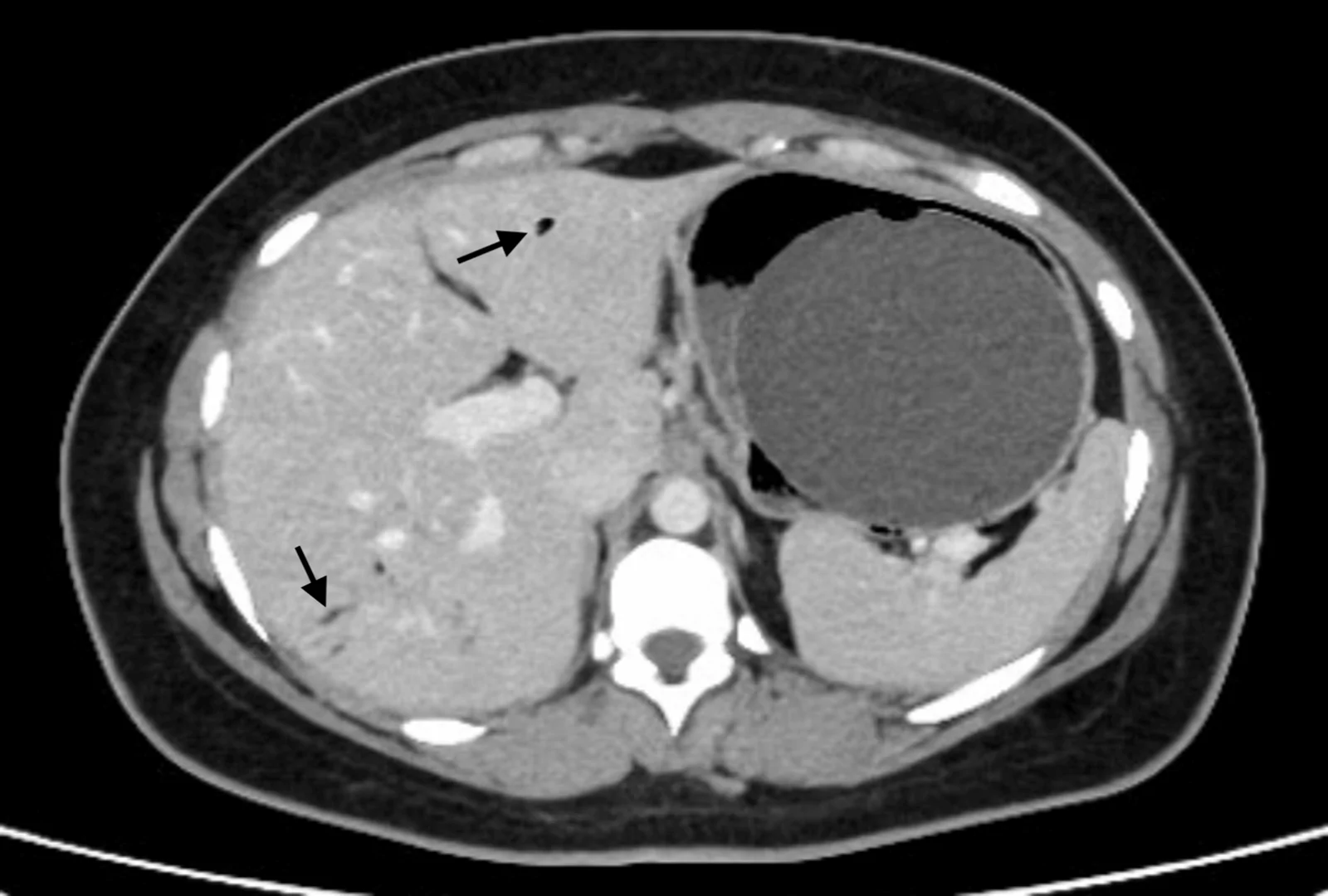
Axial section of CT scan abdomen demonstrating air within the portal venous system The arrows indicate air within the portal venous system.

The patient was made nil per os and managed with intravenous fluids, pantoprazole, analgesics, and antiemetics. Upper endoscopy revealed a well-inflated balloon with significant gastric mucosal edema, erythema, and severe erosions along the greater curvature (Figures 3, 4).

**Figure 3:**
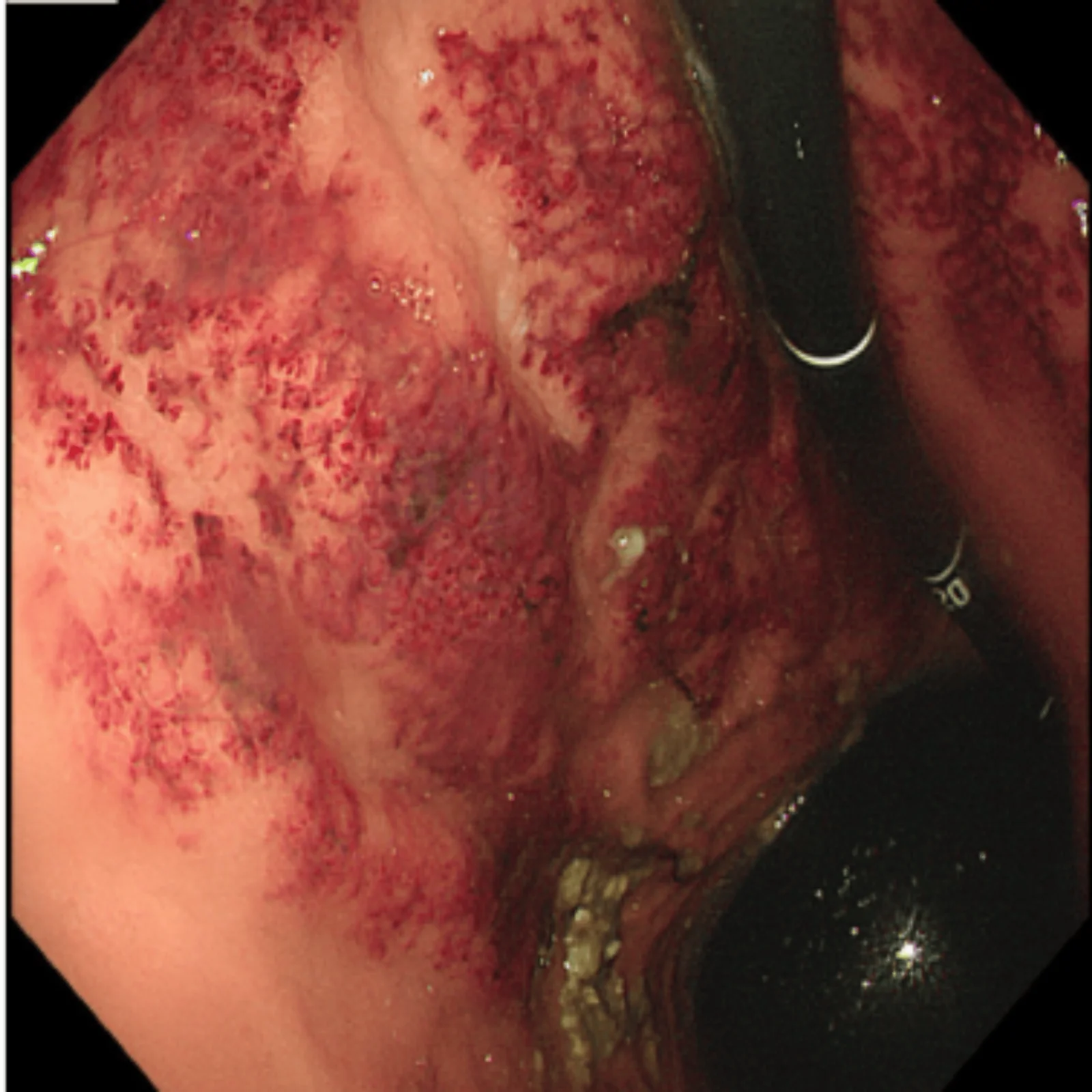
Endoscopic image in retroflexion demonstrating the intragastric balloon with surrounding gastric mucosal edema, erythema, and erosions

**Figure 4:**
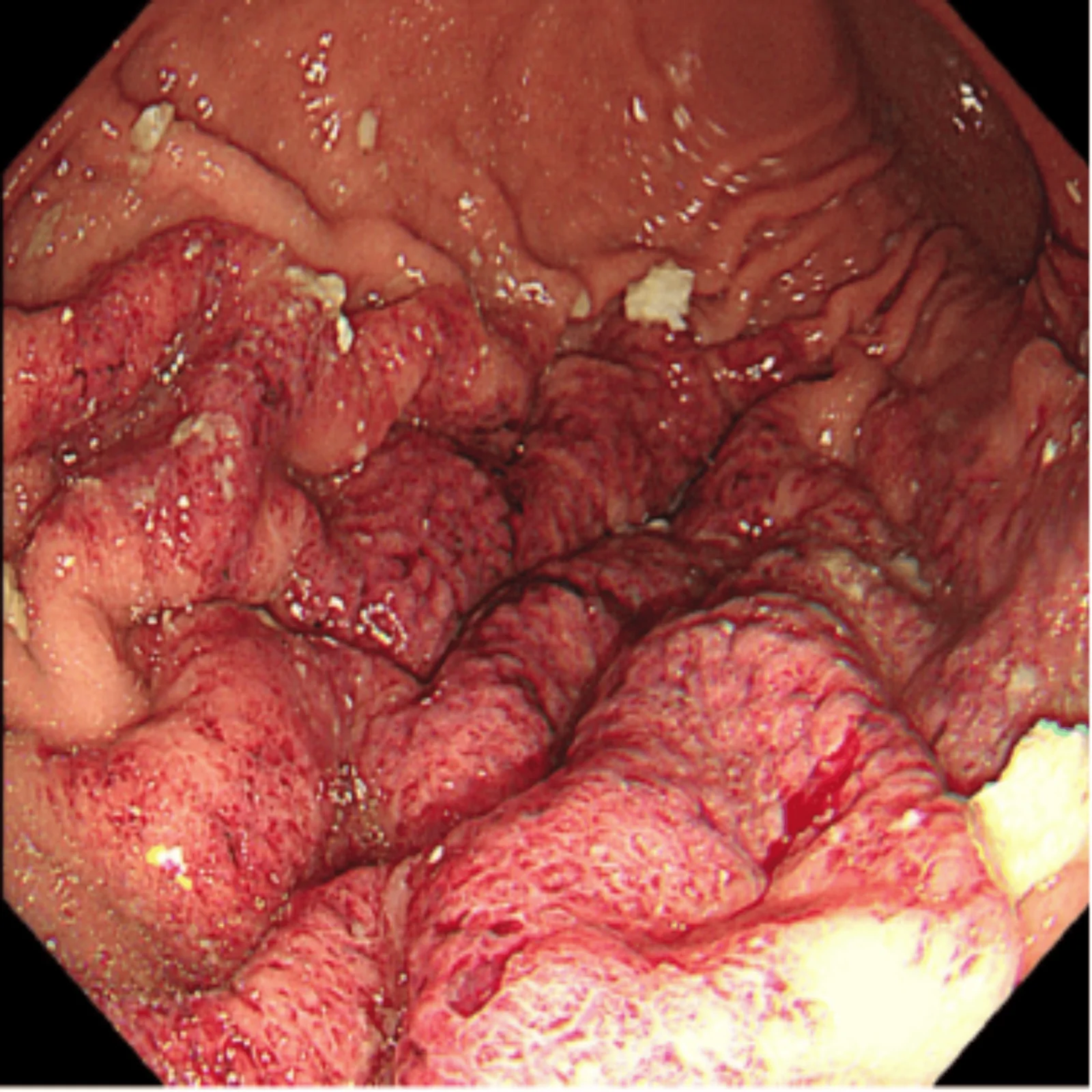
Endoscopic image demonstrating gastric mucosa with severe edema, erythema, and erosions

The balloon was aspirated and successfully removed. Post-procedure, she remained nil per os for 24 hours, after which she gradually resumed an oral diet without recurrence of symptoms. Further investigations, including serum lactate and D-dimer, were within normal limits. A repeat CT scan performed seven days later demonstrated complete resolution of HPVG (Figures 5, 6), and the patient remained clinically stable and asymptomatic.

**Figure 5:**
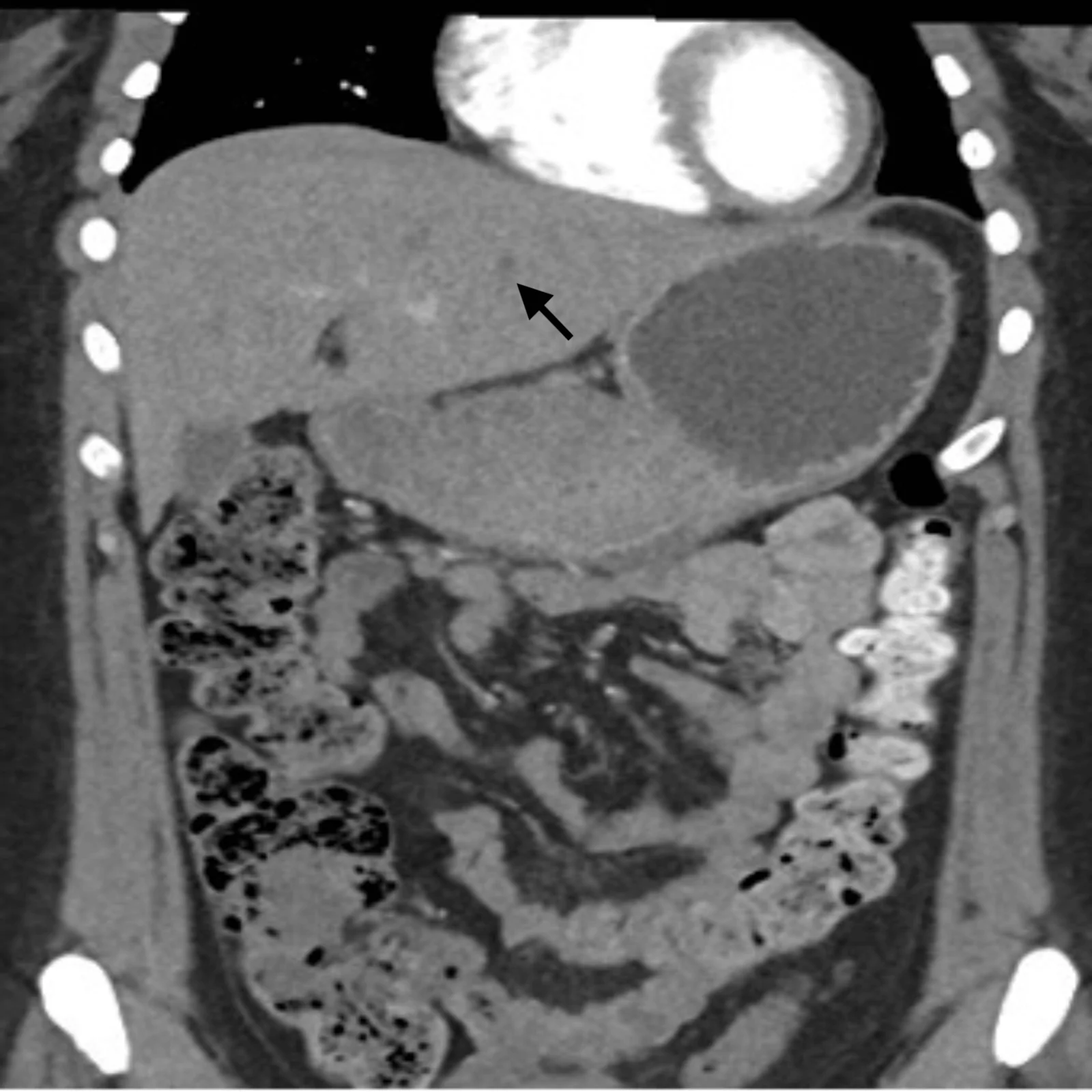
CT scan of the abdomen demonstrating complete resolution of gas in the portal venous system The arrow indicates the portal venous system without evidence of intravascular air.

**Figure 6:**
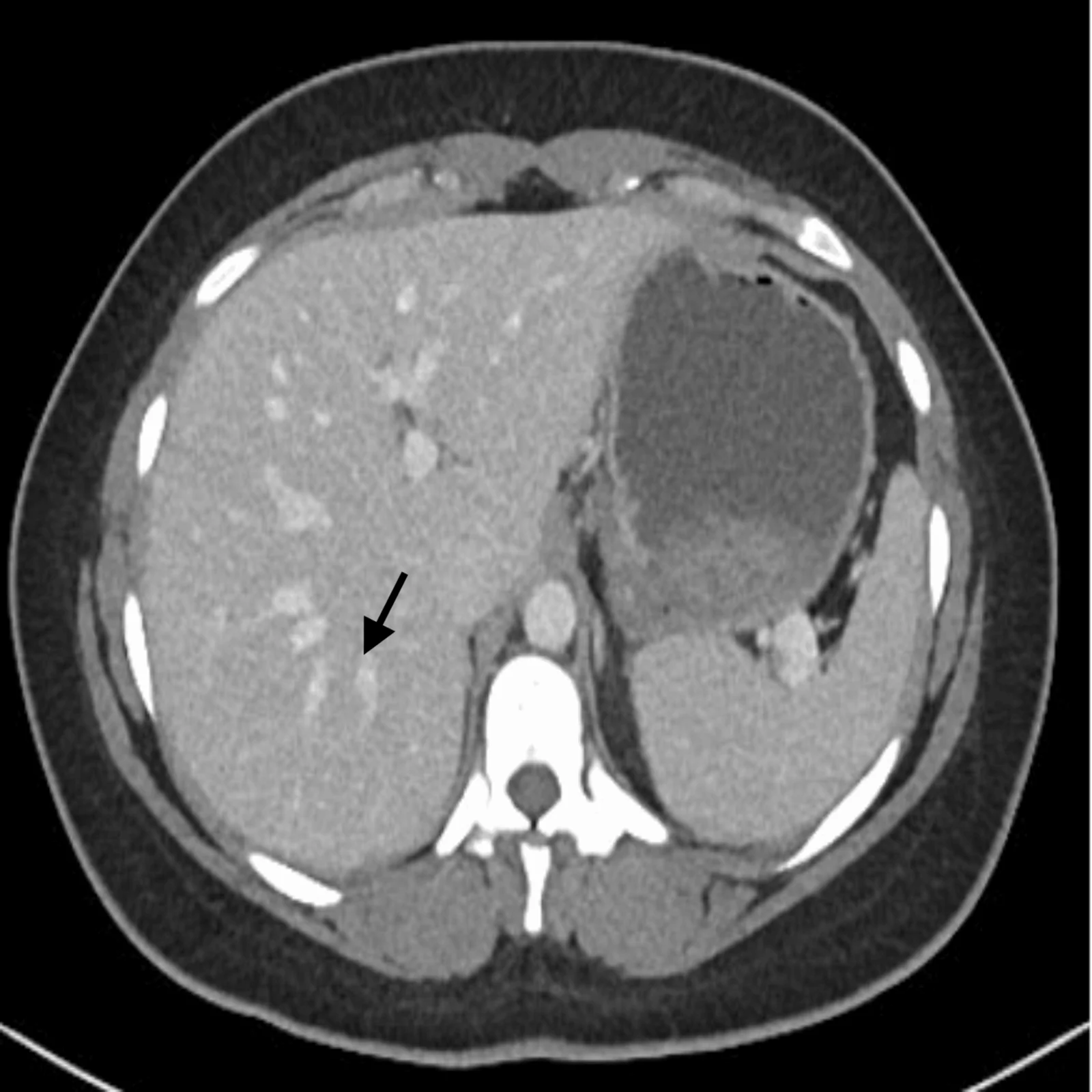
CT scan of the abdomen demonstrating resolution of gas in the portal venous system The arrow indicates the portal venous system without evidence of intravascular air.

## Discussion

IGB placement is a well-established, minimally invasive weight-loss strategy, and its adverse-event profile is well described [1-3]. Most patients have accommodative symptoms, such as nausea, vomiting, and abdominal cramping, in the first days to weeks after insertion. These usually settle with antiemetics and proton pump inhibitors. In about 7.5% of patients, however, persistent intolerance leads to early balloon removal [2]. Gastroesophageal reflux is common. Gastric erosions and ulcers, probably caused by pressure from the balloon on the gastric wall, are reported in about 2% of patients [2]. Serious complications are rare. They include balloon deflation and migration with bowel obstruction (about 1.4%), gastric perforation (about 0.1%), esophageal perforation, acute pancreatitis, and spontaneous balloon hyperinflation [1-3]. Device-, patient-, and operator-related factors, including leaving the balloon in place beyond the recommended period, have been implicated in many life-threatening events [3]. HPVG has been reported only once in a patient with an IGB [7].

HPVG was first described by Wolfe and Evans in 1955 in infants, who on autopsy were found to have necrotizing enterocolitis [8]. Three mechanisms can allow gas to enter the portal circulation: mucosal injury, raised intraluminal pressure, and infection with gas-producing bacteria [4,5]. Historically, HPVG has been associated with life-threatening conditions such as bowel ischemia, necrotizing enterocolitis, inflammatory bowel disease, and intra-abdominal sepsis [5]. Less often, it has been linked to acute necrotizing pancreatitis, massive gastric dilatation secondary to superior mesenteric artery syndrome, and percutaneous endoscopic gastrostomy [9,10].

As CT imaging has improved, it has become clear that HPVG does not always mean a catastrophic intra-abdominal process [6]. In their review, Abboud et al. cited an overall mortality of 39% in a cumulative series of 182 adult cases, lower than earlier reports. They emphasized that prognosis depends on the underlying cause rather than on HPVG itself [5].

A more recent retrospective analysis of 20 patients by Zhang et al. supported this view. Many of their HPVG cases followed a benign course and could be managed conservatively, and mortality and severity depended largely on the primary pathology and the patient's systemic status rather than on the HPVG finding itself [6].

In 2012, Vongsuvanh et al. described the only other reported case of HPVG in a patient with an IGB. Migration of a Spatz balloon catheter into the duodenum led to severe acute necrotizing pancreatitis, gastric ischemia, and HPVG, and the device had to be removed surgically. Intraoperative findings confirmed significant pancreatic inflammation, mucosal ischemia, and hemorrhage. Despite a complicated postoperative course, the patient recovered [7].

Our patient's presentation was considerably less severe. The IGB had been in place beyond the recommended duration, but the patient presented mainly with gastrointestinal symptoms after a suspected episode of acute gastroenteritis. Imaging showed HPVG without evidence of perforation or ischemia. Endoscopy showed mucosal erosions but no balloon migration. The balloon was intact and was removed electively, and the HPVG resolved completely with conservative management alone.

The HPVG in our patient was probably multifactorial, and it cannot be attributed to the IGB alone. Several recognized risk factors were present at the same time. First, repeated vomiting and gastric distension would have raised intragastric pressure, an established mechanism of non-ischemic HPVG [5,9,10]. Second, the severe erosive gastropathy seen at endoscopy offered a likely route for gas in the stomach to enter the portal venous system [5,9]. Third, the suspected acute gastroenteritis may have contributed through mucosal inflammation and possibly gas-forming organisms [4,5]. The IGB, which had stayed in place longer than recommended, may have added to these factors. It could have injured the mucosa through pressure on the gastric wall and worsened the distension and vomiting [2,3]. However, each of these factors has been reported to cause HPVG on its own, so the independent contribution of the balloon cannot be determined.

Importantly, the patient showed no signs of peritonitis, bowel ischemia, or systemic compromise. This fits with recent literature showing that HPVG can be managed without surgery once ischemia and perforation have been excluded clinically and radiologically [5,6]. In both the Abboud et al. and Zhang et al. series, some patients with benign or transient causes of HPVG, such as enteritis or upper gastrointestinal distension, recovered fully with supportive treatment alone [5,6].

In this case, conservative management led to complete resolution of symptoms. This included bowel rest, intravenous fluids, pharmacologic support, and timely removal of the balloon. The approach is consistent with evidence favoring individualized management of HPVG based on clinical and radiologic findings rather than routine aggressive intervention [5,6].

This report has limitations. Several known causes of HPVG occurred together, so we cannot establish a causal relationship between the IGB and HPVG or estimate the balloon's own contribution. Our observations describe an association and a plausible mechanism and should be considered hypothesis-generating.

## Conclusions

HPVG is an uncommon radiologic finding with a wide range of underlying causes and prognoses. This case shows that HPVG can occur in a patient with an IGB, in the setting of severe vomiting, gastric distension, and mucosal injury, and can follow a benign course. The balloon may have been one of several contributing factors, but a single case cannot establish a causal relationship. When bowel ischemia and perforation have been excluded clinically and radiologically, conservative management with removal of the balloon may be appropriate and can help avoid unnecessary surgery.
